# Clinical and imaging impact of diabetes mellitus on elderly patients with lumbar spinal stenosis: a retrospective propensity score-matched study with ≥5-year follow-up

**DOI:** 10.3389/fmed.2026.1801937

**Published:** 2026-05-29

**Authors:** Tusheng Li, Baodong Wang, Aobo Wang, Ning Fan, Ziqian Ma, Lei Zang

**Affiliations:** Department of Orthopedics, Beijing Chaoyang Hospital, Capital Medical University, Beijing, China

**Keywords:** diabetes mellitus, disc degeneration, elderly patients, lumbar spinal stenosis, propensity score matching

## Abstract

**Objective:**

Percutaneous transforaminal endoscopic decompression (PTED) is an effective minimally invasive procedure for elderly patients with lumbar spinal stenosis (LSS). This study aimed to investigate the long-term clinical and imaging effects of diabetes mellitus (DM) in elderly patients with LSS treated with PTED.

**Methods:**

From January 2016 to December 2019, this study retrospectively reviewed 232 patients with single-level LSS who underwent PTED, comprising 45 patients with DM and 187 without DM. Propensity score matching (PSM) was conducted to balance baseline covariates between the DM and non-DM groups. Demographic data, clinical scores, radiographic parameters, complications, and reoperation rates were collected and compared.

**Results:**

After PSM, 74 patients were included, with a follow-up duration of 60–108 months. Compared with the DM group, the non-DM group demonstrated significantly greater improvement in visual analog scale scores for low back pain and leg pain, Japanese Orthopaedic Association score, and Oswestry Disability Index at 24 months postoperatively and at the last follow-up (*p* < 0.05). The non-DM group demonstrated a higher ratio of greyscale value than the DM group at 24 months postoperatively and at the last follow-up (*p* < 0.05). The DM group showed a more pronounced decrease in disc height index and a higher proportion of high-grade multifidus fatty atrophy at the last follow-up (*p* < 0.05). The excellent-and-good rate based on the modified MacNab criteria at the last follow-up was higher in the non-DM group than in the DM group (*p* < 0.05). No significant differences were observed in the incidence of complications or reoperation rates between the two groups (*p* > 0.05).

**Conclusion:**

Elderly patients with LSS and DM demonstrated poorer long-term clinical outcomes after PTED. DM was associated with more severe intervertebral disc degeneration and greater fat infiltration of the multifidus muscles. Stricter postoperative glycemic control and comprehensive management of muscle function are required for elderly patients with LSS and DM.

## Introduction

Lumbar spinal stenosis (LSS) is a prevalent degenerative spinal disorder, particularly in the elderly population (1). It is characterized by central canal narrowing, lateral recess, and/or intervertebral foramina secondary to pathologies, such as disc herniation, ligamentum flavum hypertrophy, and facet joint osteophytes, leading to nerve roots and/or cauda equina compression and causing a clinical syndrome typified by neurogenic intermittent claudication (1, 2). Surgery is the mainstay of management for patients who fail conservative treatment.

Conventional open decompression achieves sufficient neural decompression but requires extensive detachment of paraspinal muscles and wide bone resection, which leads to postoperative residual low back pain, paraspinal musculature denervation, and segmental instability, thereby adversely affecting prognosis (3, 4). With the development of minimally invasive concepts and techniques, minimally invasive spinal surgery has been increasingly applied in lumbar degenerative diseases, and spinal endoscopy has become one of the mainstream surgical options for LSS (5, 6). Several studies have revealed that percutaneous transforaminal endoscopic decompression (PTED) via the transforaminal approach provides satisfactory clinical outcomes in patients with LSS (7, 8).

Diabetes mellitus (DM) is a chronic systemic disease that affects bone, cartilage, peripheral nerves, blood vessels, and intervertebral discs. Further, its prevalence increases progressively in individuals over 60 years of age (9, 10). DM is considered an important factor that promotes lumbar degenerative disease progression (9, 10). By 2045, the global number of patients with DM is estimated to reach 783.2 million, accounting for approximately 12.5% of the world’s population (11). Previous studies have revealed DM as a risk factor for LSS and is associated with higher postoperative reoperation and complication rates after lumbar surgery (12–14).

The effect of hyperglycemia on LSS has long been a focus of clinical attention; however, studies that specifically investigate the influence of DM on postoperative outcomes in LSS remain limited. Udby et al. (10) reported that patients with LSS and DM experienced more residual leg pain at 2-year follow-up postoperatively than those without DM. Arinzon et al. (14) followed at least 65-year-old patients with or without DM for a mean of 41 months and revealed less pain relief and a higher incidence of complications in the DM group, but postoperative imaging changes were not reported. Furthermore, few studies have investigated the long-term outcomes of patients with LSS and DM after endoscopic surgery.

Elderly patients with LSS frequently present with more severe degenerative changes and a higher burden of comorbidities, leading to considerable heterogeneity in postoperative outcomes (2, 10, 15). Therefore, understanding the specific effect of DM on surgical results in this population is of great clinical importance.

In this study, we investigated the effects of DM on long-term clinical and imaging outcomes, complications, and reoperation rates in elderly patients with LSS who underwent PTED, with a minimum follow-up of 5 years. Meanwhile, a scoring-matching approach was employed to balance potential confounders between groups and improve the reliability of our findings.

## Materials and methods

### Study design and patients

The Ethics Committee of Beijing Chaoyang Hospital, Capital Medical University (No. 2021-KE-478), approved this retrospective study, conducted in adherence to the Declaration of Helsinki. All participants signed written informed consent for surgery preoperatively. The requirement for written informed consent to participate was waived by the Ethics Committee because of the retrospective nature of the study.

Inclusion criteria were (1) age of ≥ 65 years; (2) diagnosis of single-level LSS; (3) failure of conservative treatment; and (4) presence of low back pain and radiating leg pain preoperatively. Exclusion criteria were (1) lumbar instability, severe spinal deformity, spinal tumor, or infection; (2) previous lumbar surgery; (3) severe systemic diseases, such as hepatic or renal failure; (4) psychiatric disorders; (5) pregnancy; and (6) incomplete follow-up data.

Patients were categorized based on the presence of diabetes into the DM and non-DM groups. DM was diagnosed according to the American Diabetes Association criteria (16). This study included 232 patients, comprising 45 in the DM group and 187 in the non-DM group, from January 2016 to December 2019.

**Figure 1:**
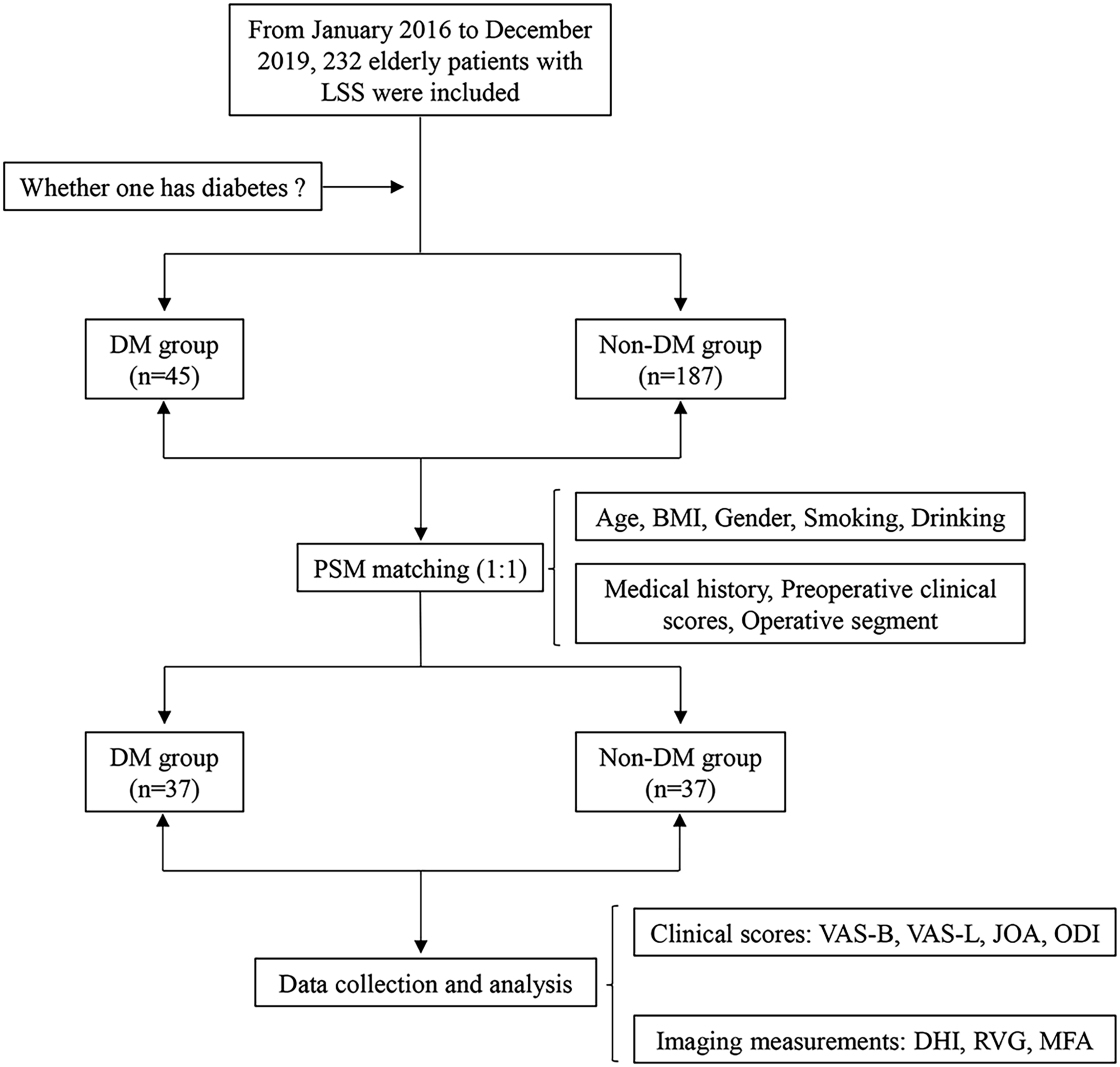
Research design flowchart.

Propensity score matching (PSM) was employed to adjust for baseline imbalances between the two groups. The covariates entered into the PSM model included (1) age; (2) body mass index (BMI); (3) gender; (4) medical history; (5) preoperative clinical scores; (6) operative segment; (7) smoking history; and (8) drinking history. Figure 1 illustrates the research design flowchart.

### Surgical procedure

A single senior spine surgeon experienced in endoscopic techniques performed all PTED procedures under local anesthesia. Patients were placed in the prone position. The operative level was confirmed under fluoroscopic guidance. The entry point was located approximately 10–14 cm lateral to the midline, and a transforaminal approach was employed. Under fluoroscopy, a guidewire was placed at the tip of the superior articular process (SAP) of the responsible segment via a puncture needle. A skin incision of approximately 1 cm was created, and sequential dilators were used to dilate the soft tissues, followed by working cannula insertion. A trephine and/or endoscopic grinding drill were used to partially resect the SAP to achieve foraminoplasty depending on intraoperative requirements. Hypertrophic ligamentum flavum and herniated disc material were removed under endoscopic visualization. The compressed nerve root and dural sac were carefully explored and decompressed. When the nerve root was relaxed with visible pulsation and hyperemic vessels on its surface, adequate decompression was confirmed. Hemostasis was achieved, the working channel was removed, and the incision was closed.

### Follow-up and data collection

Demographic data of all successfully matched elderly patients with LSS were collected. Patients were followed up by outpatient visits, telephone, or e-mail. The minimum follow-up duration was 5 years. Clinical scores, imaging parameters, complications, and reoperation information were recorded during the follow-up period. For patients with DM, diabetes-related characteristics, including HbA1c level, duration of diabetes, diabetes classification, antidiabetic treatment, and diabetes-related complications, were also collected.

### Clinical evaluation

Self-administered questionnaires, including visual analog scale scores for low back pain (VAS-B) and leg pain (VAS-L), Japanese Orthopaedic Association (JOA) score, and Oswestry Disability Index (ODI), were used to evaluate clinical outcomes. At the last follow-up, patient satisfaction was assessed following the modified MacNab criteria, which categorize outcomes as excellent, good, fair, or poor. The excellent-and-good rate was calculated as follows: (number of excellent + number of good) / total number of patients × 100% (17).

### Imaging measurements

All patients underwent lumbar radiography and magnetic resonance imaging (MRI) preoperatively, at 24 months postoperatively, and at the last follow-up. Imaging parameters included disc height index (DHI), ratio value of greyscale (RVG), and degree of multifidus fatty atrophy (MFA). Two observers independently performed all imaging measurements, and the mean values were used for analysis.

**Figure 2:**
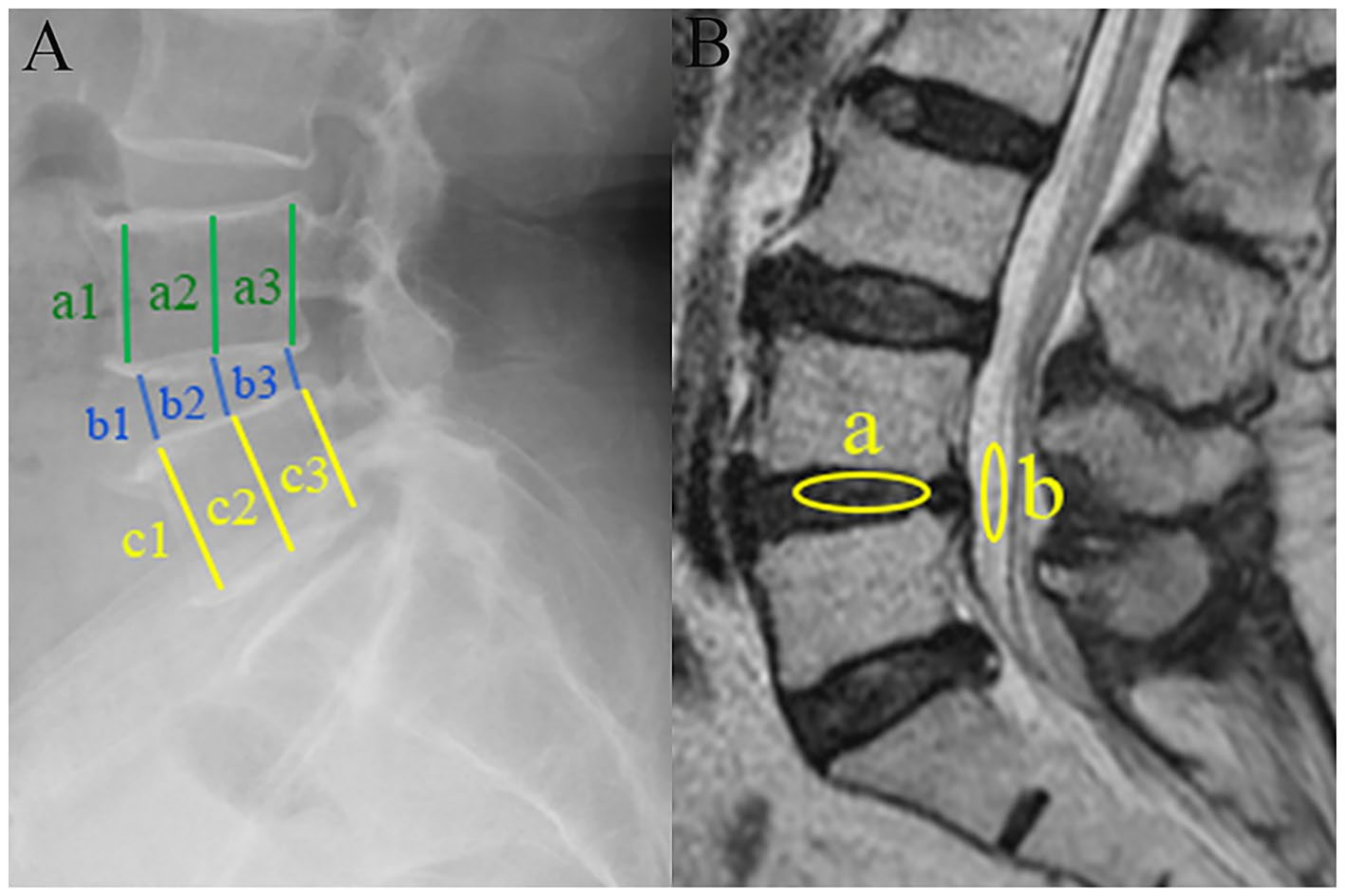
Schematic diagram of DHI and RVG measurements. (A) DHI = [2 × (b1 + b2 + b3)] / [(a1 + a2 + a3) + (c1 + c2 + c3)] × 100%. RVG = average disc greyscale value (a)/average cerebrospinal fluid greyscale value (b) × 100%.

According to previous literature (18), the heights of the anterior, middle, and posterior margins of the intervertebral disc and adjacent vertebral bodies were measured separately, and the average values were calculated to minimize measurement errors. DHI = [2 × (sum of intervertebral disc heights)] / [(sum of superior vertebral body heights) + (sum of inferior vertebral body heights)] × 100% (Figure 2A).

RVG was defined as the ratio of the mean greyscale value of the disc to that of the cerebrospinal fluid within the same segment, expressed as follows: RVG = average disc greyscale value / average cerebrospinal fluid greyscale value × 100% (18) (Figure 2B).

The area of the multifidus muscle and fat infiltration were measured following the method reported by Zhu et al. (19). The degree of MFA was graded according to the proportion of fat infiltration in the multifidus muscle: 0–10% (normal), 10–30% (mild), 30–50% (moderate), and >50% (severe). Normal to mild MFA was defined as low-grade MFA, and moderate to severe MFA as high-grade MFA (20).

### Statistical analysis

To minimize potential selection bias and baseline confounding between the DM and non-DM groups, PSM was performed using IBM SPSS Statistics version 25.0. Propensity scores were estimated for each patient using a logistic regression model, with diabetes status as the dependent variable and age, BMI, gender, medical history, preoperative clinical scores, operative segment, smoking history, and drinking history as covariates. Patients in the DM group were matched 1:1 with patients in the non-DM group using a caliper-based matching algorithm without replacement, with a caliper width of 0.02 on the propensity score scale. After matching, baseline characteristics were compared between the two groups to assess covariate balance.

Continuous variables with normal distribution were compared using an independent-sample *t*-test and presented as mean ± standard deviation. Non-parametric tests were used to analyze non-normally distributed continuous variables, which were presented as median (Q1, Q3). The Chi-square test or Fisher’s exact test was used to compare categorical variables, which were expressed as frequencies and percentages. Non-parametric tests for related samples and repeated measures analysis of variance were used for within-group comparisons at different time points. In the matched DM group, exploratory correlation analyses were performed to evaluate the associations between HbA1c level and changes in VAS-B, VAS-L, ODI, and DHI from baseline to the last follow-up. Spearman rank correlation analysis was used for the associations of HbA1c level with changes in VAS-B, VAS-L, and DHI, whereas Pearson correlation analysis was used for the association between HbA1c level and change in ODI. A *p*-value of <0.05 indicated statistical significance.

## Results

### Baseline characteristics before and after PSM

Table 1 summarizes the general information of patients before PSM. Before matching, the two groups demonstrated imbalances in several preoperative clinical scores, including VAS-B, VAS-L, JOA, and ODI. After PSM, 74 patients (37 in each group) were included, and all covariates were well balanced between the DM and non-DM groups (Table 2). The mean follow-up duration was 79.22 ± 12.28 months (range, 60–103 months) and 83.84 ± 14.65 months (range, 60–108 months) in the DM and non-DM groups, respectively, with no significant difference between groups (*p* = 0.146). In addition, diabetes-related characteristics of the matched DM group, including HbA1c level, duration of diabetes, diabetes classification, antidiabetic treatment, and specific diabetes-related complications, are presented in Supplementary Table S1. Exploratory correlation analyses within the matched DM group showed that HbA1c level was positively correlated with the change in DHI from baseline to the last follow-up (*ρ* = 0.446, *p* = 0.006), whereas no significant correlations were observed between HbA1c level and changes in VAS-B, VAS-L, or ODI (Supplementary Table S2).

**Table 1:** General information before PSM.

Variable	DM group (*n* = 45)	Non-DM group (*n* = 187)	*p*-value
Age (years)	71.00 (68.00, 78.00)	73.00 (68.00, 78.00)	0.445
BMI (kg/m^2^)	25.39 (23.52, 27.15)	25.61 ± 3.68	0.904
Gender, *n* (%)			0.175
Male	26 (57.78)	87 (46.52)	
Female	19 (42.22)	100 (53.48)	
Medical history, *n* (%)			
Hypertension	21 (46.67)	80 (42.78)	0.637
Heart disease	4 (8.89)	30 (16.04)	0.223
Preoperative clinical scores			
VAS-B score	5 (4, 5.5)	4 (4, 5)	0.027
VAS-L score	7 (6, 7)	6 (6, 7)	0.018
JOA score	13.44 ± 1.94	14 (13, 15)	0.004
ODI (%)	42.67 ± 7.85	40 (36, 44)	0.012
Operative segment, *n* (%)			0.437
L3-4	4 (8.89)	17 (9.09)	
L4-5	31 (68.89)	143 (76.47)	
L5-S1	10 (22.22)	27 (14.44)	
Smoking, *n* (%)	11 (24.44)	47 (25.13)	0.924
Drinking, *n* (%)	5 (11.11)	30 (16.04)	0.407

**Table 2:** General information after PSM.

Variable	DM group (*n* = 37)	Non-DM group (*n* = 37)	*p*-value
Age (years)	73.00 (69.00, 79.00)	72.00 (66.00, 76.00)	0.509
BMI (kg/m^2^)	25.34 (23.42, 26.66)	25.85 ± 3.80	0.452
Gender, *n* (%)			0.816
Male	19 (51.35)	18 (48.65)	
Female	18 (48.65)	19 (51.35)	
Medical history, *n* (%)			
Hypertension	16 (43.24)	15 (40.54)	0.814
Heart disease	4 (10.81)	1 (2.70)	0.354
Preoperative clinical scores			
VAS-B score	4 (4, 5)	5 (4, 5)	0.756
VAS-L score	6 (6, 7)	6 (6, 7)	0.955
JOA score	13.92 ± 1.75	13.95 ± 1.82	0.948
ODI (%)	40.70 ± 6.64	40.86 ± 7.57	0.922
Operative segment, *n* (%)			0.254
L3-4	4 (10.81)	3 (8.11)	
L4-5	24 (64.86)	30 (81.08)	
L5-S1	9 (24.32)	4 (10.81)	
Smoking, *n* (%)	9 (24.32)	11 (29.73)	0.601
Drinking, *n* (%)	4 (10.81)	4 (10.81)	1.000

### Clinical outcomes

**Figure 3:**
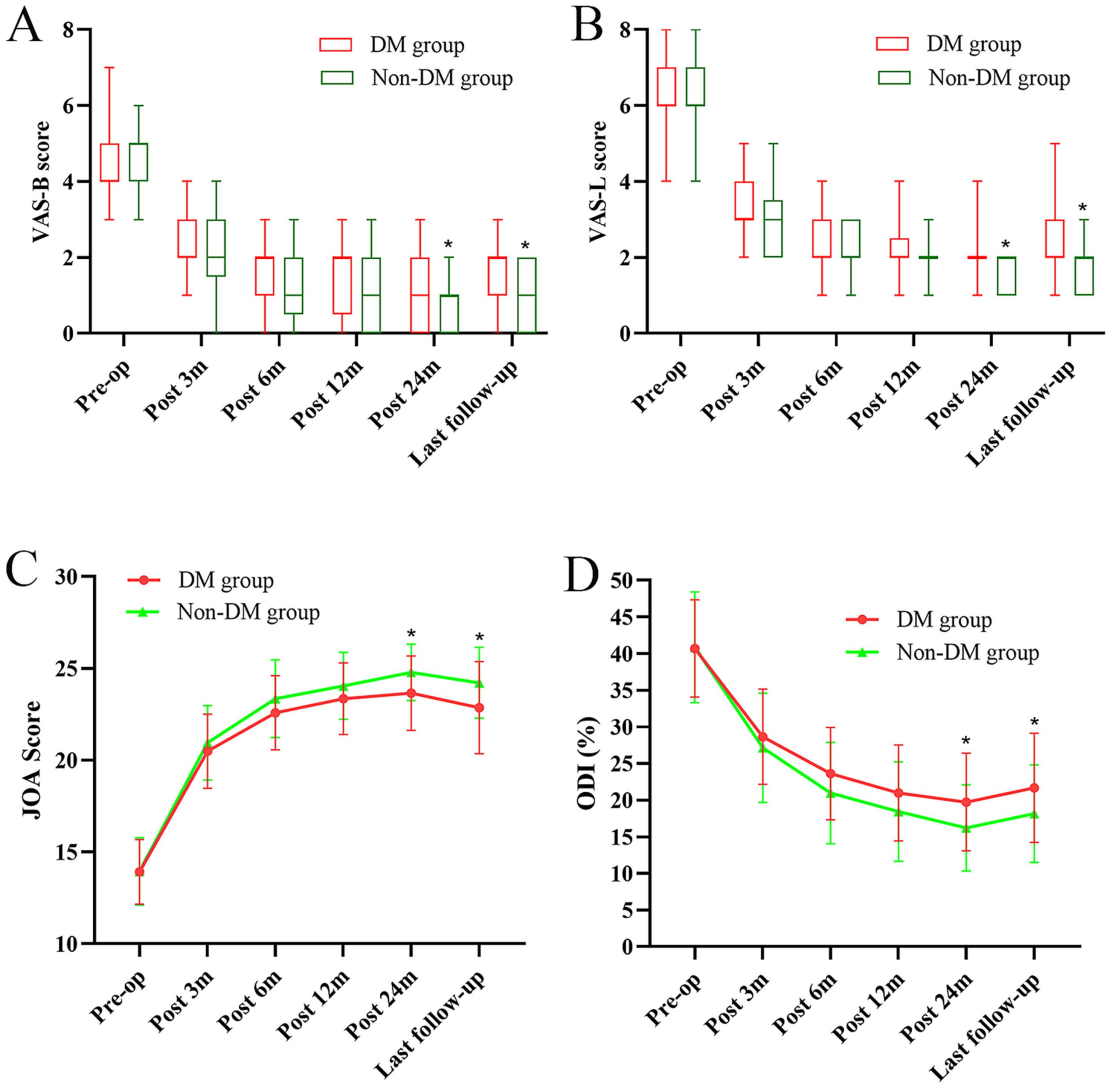
Schematic diagram of clinical outcomes. **(A)** Changes in VAS-B scores. **(B)** Changes in VAS-L scores. **(C)** Changes in JOA scores. **(D)** Changes in ODI scores. * Statistically significant differences between groups.

Table 3 and Figure 3 show the clinical outcomes of both groups. Preoperatively, no significant differences were observed between the DM and non-DM groups in clinical functional scores (*p* > 0.05). In both groups, postoperative VAS-B, VAS-L, JOA, and ODI demonstrated significant improvement compared with baseline (*p* < 0.05). At 24 months postoperatively and at the last follow-up, the non-DM group demonstrated significantly greater improvements in VAS-B, VAS-L, JOA, and ODI than the DM group (*p* < 0.05), indicating superior pain relief and functional recovery in patients without DM.

**Table 3:** Clinical outcomes in both groups.

Clinical outcome/time point	DM group (*n* = 37)	Non-DM group (*n* = 37)	*p*-value
VAS-B score, M (Q1, Q3)			
Pre-op	4 (4, 5)	5 (4, 5)	0.756
Post 3 m	2 (2, 3)*	2 (1.5, 3)*	0.308
Post 6 m	2 (1, 2)*	1 (0.5, 2)*	0.113
Post 12 m	2 (0.5, 2)*	1 (0, 2)*	0.130
Post 24 m	1 (0, 2)*	1 (0, 1)*	0.037
Last follow-up	2 (1, 2)*	1 (0, 2)*	0.030
VAS-L score, M (Q1, Q3)			
Pre-op	6 (6, 7)	6 (6, 7)	0.955
Post 3 m	3 (3, 4)*	3 (2, 3.5)*	0.128
Post 6 m	2 (2, 3)*	2 (2, 3)*	0.087
Post 12 m	2 (2, 2.5)*	2 (2, 2)*	0.098
Post 24 m	2 (2, 2)*	2 (1, 2)*	0.043
Last follow-up	2 (2, 3)*	2 (1, 2)*	0.034
JOA score, Mean ± SD			
Pre-op	13.92 ± 1.75	13.95 ± 1.82	0.948
Post 3 m	20.49 ± 2.02*	20.95 ± 2.03*	0.332
Post 6 m	22.59 ± 2.02*	23.35 ± 2.12*	0.121
Post 12 m	23.35 ± 1.95*	24.05 ± 1.82*	0.114
Post 24 m	23.65 ± 2.03*	24.78 ± 1.53*	0.008
Last follow-up	22.86 ± 2.51*	24.22 ± 1.93*	0.011
ODI (%), Mean ± SD			
Pre-op	40.70 ± 6.64	40.86 ± 7.57	0.922
Post 3 m	28.65 ± 6.50*	27.14 ± 7.45*	0.355
Post 6 m	23.62 ± 6.29*	20.97 ± 6.91*	0.089
Post 12 m	20.97 ± 6.54*	18.43 ± 6.78*	0.105
Post 24 m	19.73 ± 6.67*	16.22 ± 5.88*	0.019
Last follow-up	21.68 ± 7.46*	18.16 ± 6.66*	0.036

* Compared with the preoperative value, *p* < 0.05.

At the last follow-up, according to the modified MacNab criteria, outcomes in the non-DM group were excellent in 26 patients, good in 6, fair in 4 and poor in 1. In the DM group, outcomes were excellent in 14 patients, good in 10, fair in 9 and poor in 4. The excellent-and-good rate was 86.49% (32/37) and 64.86% (24/37) in the non-DM and DM groups, respectively, with a statistically significant difference (*p* = 0.030) (Table 4).

**Table 4:** Modified MacNab criteria, complications and reoperation rate in both groups.

Item	DM group (*n* = 37)	Non-DM group (*n* = 37)	*p*-value
MacNab evaluation			0.030
Excellent + good	24	32	
Fair + poor	13	5	
Complications			
Dural sac tear	4	2	
Epidural hematoma	1	0	
Nerve root injury	0	0	
Wound infection	0	0	
Intervertebral disc Infection	0	0	
Total number	5	2	0.427
Reoperation	4	1	0.354

### Imaging outcomes

**Figure 4:**
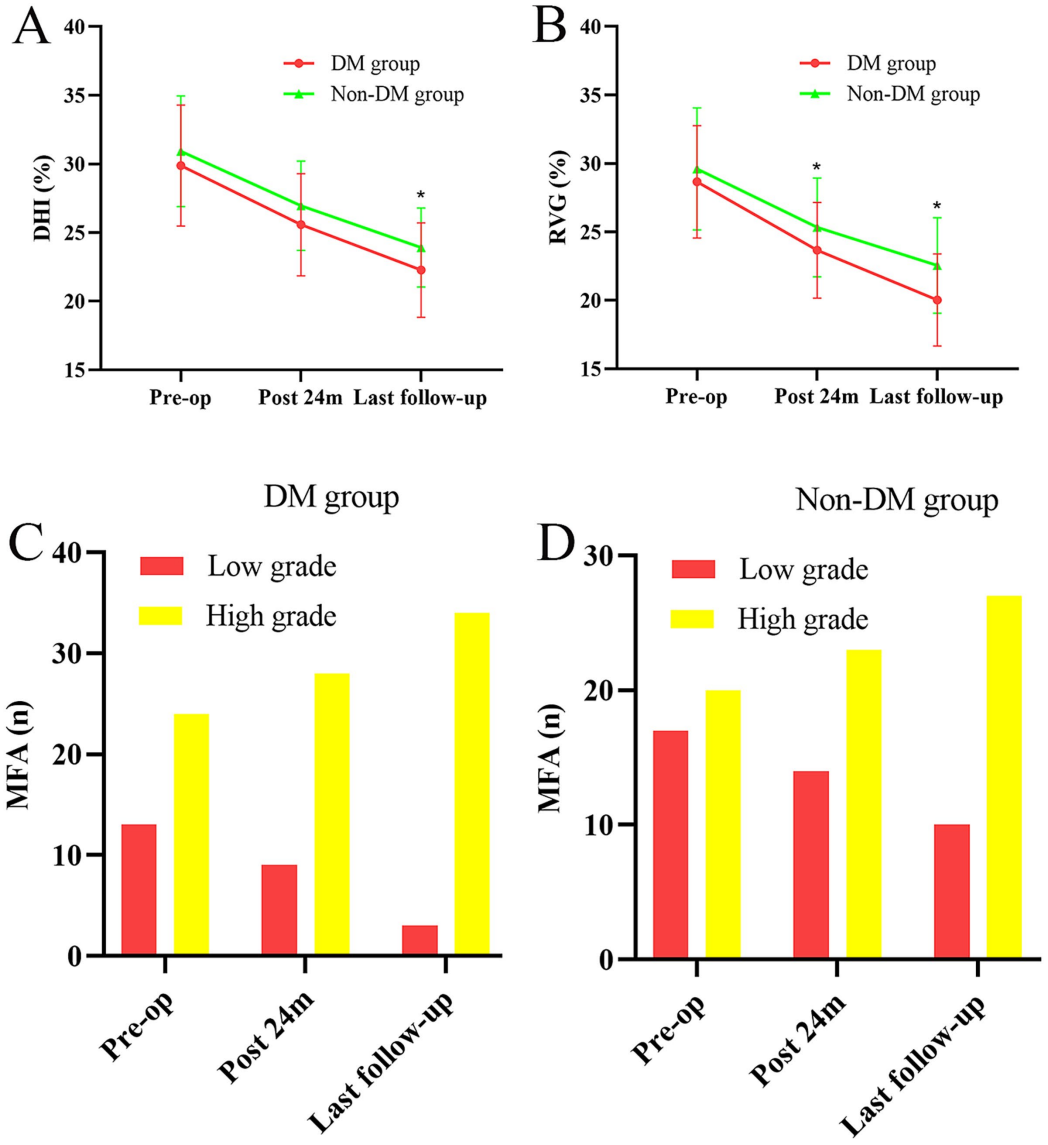
Schematic diagram of imaging outcomes. **(A)** Changes in DHI. **(B)** Changes in RVG. **(C)** Changes in MFA in the DM group. **(D)** Changes in MFA in the non-DM group. * Statistically significant differences between groups.

Table 5 and Figure 4 present imaging outcomes. Preoperatively, no significant differences were observed between the two groups in DHI, RVG, or MFA proportion (*p* > 0.05). At 24 months postoperatively and at the last follow-up, RVG reduction was significantly greater in the DM group than in the non-DM group (*p* < 0.05). Further, at the last follow-up, the DM group showed a more pronounced decrease in DHI and a higher proportion of high-grade MFA than the non-DM group (*p* < 0.05). These findings indicate that DM is associated with more severe disc degeneration and more advanced multifidus fatty infiltration over the long term in elderly patients with LSS after PTED.

**Table 5:** Imaging outcomes in both groups.

Imaging parameter/time point	DM group (*n* = 37)	Non-DM group (*n* = 37)	*p*-value
DHI (%), Mean ± SD			
Pre-op	29.88 ± 4.40	30.92 ± 4.03	0.292
Post 24 m	25.57 ± 3.72	26.95 ± 3.25	0.094
Last follow-up	22.26 ± 3.44	23.91 ± 2.87	0.028
RVG (%), Mean ± SD			
Pre-op	28.65 ± 4.10	29.60 ± 4.46	0.344
Post 24 m	23.66 ± 3.49	25.33 ± 3.61	0.048
Last follow-up	20.02 ± 3.36	22.54 ± 3.49	0.002
MFA, (Low/High grade), *n*			
Pre-op	13/24	17/20	0.344
Post 24 m	9/28	14/23	0.209
Last follow-up	3/34	10/27	0.032

### Complications and reoperation rates

In terms of complications, 4 dural sac tear cases and 1 epidural hematoma case were reported in the DM group, and 2 dural sac tear cases in the non-DM group. The incidence of complications was 13.51% (5/37) and 5.41% (2/37) in the DM and non-DM groups, respectively, with no significant difference between groups (*p* = 0.427). Four and one patients in the DM and non-DM groups, respectively, underwent reoperation due to recurrent LSS, yielding reoperation rates of 10.81% and 2.70%, respectively. The difference in reoperation rates did not reach statistical significance (*p* = 0.354) (Table 4).

## Discussion

With the advancement of minimally invasive spinal techniques, endoscopic surgery has become an effective alternative to open decompression for treating LSS, providing the advantages of smaller incisions, less soft-tissue trauma, faster recovery, and shorter hospital stay (5). PTED via the transforaminal approach has provided satisfactory clinical outcomes (7, 8), even in elderly patients with LSS (21). However, elderly individuals present substantial heterogeneity due to a high prevalence of comorbidities, which may affect postoperative results (10, 15). Among these comorbidities, DM is considered one of the most important factors influencing surgical outcomes in elderly patients with LSS (9, 10, 12–14).

The incidence of DM among patients with LSS is higher than that in the general population (22). Multiple studies have indicated that DM may increase the risk of poor postoperative outcomes, including higher reoperation rates, prolonged hospital stay, increased medical costs, and a higher surgical site infection incidence (12–14, 23). Nagata et al. (24) involved 993 patients and revealed that DM was associated with worse leg pain and poorer patient-reported outcomes 1 year after lumbar surgery. Shi et al. (9) reported that patients with LSS and type 2 DM experienced less improvement in leg pain within 12 weeks after a 6-week therapeutic exercise program. However, evidence regarding the long-term effects of DM on postoperative outcomes in LSS remains limited, and imaging changes have rarely been analyzed. Experimental studies have revealed that DM leads to various pathological changes in spinal structures, including decreased disc height, reduced vertebral bone mass, and endplate sclerosis (12, 25, 26). These findings highlight the importance of investigating the imaging effect of DM in clinical studies of LSS surgery. Therefore, this study conducted a minimum 5-year follow-up of elderly patients with LSS with or without DM after PTED and assessed the effect of DM on clinical outcomes, imaging parameters, complications, and reoperation rates. To minimize confounding bias, PSM was used to match major demographic and clinical variables between groups.

In the present study, we revealed that elderly patients with LSS and DM demonstrated significantly poorer long-term clinical improvements in pain and function compared with those without DM. From an imaging perspective, the DM group demonstrated a more pronounced decrease in RVG and DHI and a higher proportion of high-grade MFA during the follow-up, indicating that DM was associated with more severe disc degeneration and multifidus fatty infiltration. The inferior clinical and imaging outcomes in patients with DM may be attributable to several mechanisms. First, diabetic neuropathy and ischemic vulnerability: Diabetic neuropathy increases the sensitivity of nerve roots to ischemic changes, making them more prone to edema; thus, even mild traction or compression of the nerve root may induce obvious radicular pain and limit postoperative functional recovery (12, 27, 28). Second, ligamentum flavum hypertrophy and mechanical compression: Type 2 DM, the most prevalent form in the elderly, is characterized by insulin resistance, which has been identified as a risk factor for ligamentum flavum hypertrophy (22). Ligamentum flavum fibrosis, loss of elastic fibers, and thickening exacerbate mechanical compression of the nerve roots, thereby worsening pain and neurological symptoms (22, 29, 30). Third, DM may exert adverse effects on intervertebral discs. Chronic hyperglycemia and advanced glycation end-product (AGE) accumulation exert deleterious effects on the intervertebral discs, including cell senescence, apoptosis, pyroptosis, oxidative stress, increased inflammatory mediators, extracellular matrix degradation, and endplate cartilage damage (31–33). These changes may contribute to disc degeneration, reduced disc height, and impaired disc mechanical properties, thereby ultimately altering spinal biomechanics (31, 32). In the exploratory correlation analysis within the matched DM group, higher HbA1c level was associated with greater loss of DHI, but not with changes in VAS-B, VAS-L, or ODI. This finding may provide preliminary quantitative support for a potential association between glycemic control and long-term disc height loss; however, it should be interpreted cautiously because of the small sample size and exploratory nature of the analysis. Fourth, DM may be associated with paraspinal muscle degeneration and fatty infiltration through both direct metabolic effects and indirect disuse-related mechanisms. DM-related microangiopathy, chronic low-grade inflammation, impaired mitochondrial function, and reduced skeletal muscle regenerative capacity may contribute to diabetic myopathy and promote multifidus fatty infiltration (31, 34–37). Meanwhile, poorer pain relief and functional recovery in the DM group may have reduced postoperative activity levels, thereby aggravating disuse-related paraspinal muscle atrophy and fatty degeneration (38). Although the present study could not determine the relative contribution of these two mechanisms, the increased MFA observed in patients with DM may reflect the combined influence of diabetes-related metabolic impairment and a secondary “pain–disuse–atrophy” cycle. Tian et al. (39) revealed that uncontrolled long-term hyperglycemia may induce lumbar disc degeneration, cause fat infiltration of paraspinal muscles at the lower lumbar levels, and increase the incidence of endplate cartilage lesions in patients with degenerative disc disease, supporting our imaging findings.

Interestingly, previous studies reported higher complication and reoperation rates in patients with LSS and DM compared with patients with no DM (12–14); however, we did not observe significant differences between groups after PSM in the present study. Several factors may explain this discrepancy. First, the relatively small sample size after matching may have limited the statistical power to detect differences. Second, strict matching may have attenuated the combined effect of DM and other high-risk factors. Third, with improvements in endoscopic techniques and instruments, decompression in PTED has become more precise, possibly decreasing the baseline incidence of complications and reoperations and thereby reducing the detectable effect of DM.

This study has several limitations. First, this was a retrospective single-center study; therefore, selection bias could not be completely eliminated, although PSM was used to reduce baseline differences between groups. In addition, residual confounding from measured or unmeasured variables may still have remained after matching, and more comprehensive balance diagnostics, such as standardized mean differences and/or graphical balance assessment, may be useful for further evaluating covariate balance. Second, the sample size after matching was relatively small, which may affect the generalizability and statistical robustness of the results. Third, although diabetes-related characteristics were summarized for patients with DM, DM was still analyzed as a binary variable in the main comparison. Owing to the relatively small number of patients in the DM group, further stratified analyses according to HbA1c level, diabetes duration, diabetes classification, antidiabetic treatment, or diabetes-related complications were not performed. Therefore, the potential influence of diabetes severity, treatment heterogeneity, and complication status on postoperative clinical and imaging outcomes could not be fully assessed. Future multicenter prospective studies with larger sample sizes and more detailed metabolic and muscle assessments are warranted to further clarify the effect of DM on clinical and imaging outcomes, complications, and reoperation rates after LSS surgery. Such evidence will help optimize perioperative glycemic and muscle function management and improve long-term prognosis in elderly patients with LSS and DM.

## Conclusion

Elderly patients with LSS and DM demonstrate poorer clinical outcomes after PTED. DM was associated with more severe intervertebral disc degeneration, greater loss of disc height, and increased fatty infiltration of the multifidus muscles. These findings indicate the need for spine surgeons to recognize DM as an important modifier of long-term outcomes after endoscopic decompression among elderly patients. Integrating strict postoperative glycemic control with targeted rehabilitation programs aimed at preserving paraspinal muscle quality may be considered in the comprehensive management of elderly patients with LSS and DM.

## Data Availability

The raw data supporting the conclusions of this article will be made available by the authors on reasonable request.

## References

[1] Lurie J Tomkins-Lane C. Management of lumbar spinal stenosis. BMJ. (2016) 352:h6234. doi: 10.1136/bmj.h6234, 26727925 PMC6887476

[2] Zaina F Tomkins-Lane C Carragee E Negrini S. Surgical versus non-surgical treatment for lumbar spinal stenosis. Cochrane Database Syst Rev. (2016) 2016:D10264. doi: 10.1002/14651858.CD010264.pub2, 26824399 PMC6669253

[3] Seo JH Park G Ju CI Kim SW Lee SM. Radiological analysis of symptomatic complications after bilateral laminotomy for lumbar spinal stenosis. Korean J Spine. (2012) 9:18–23. doi: 10.14245/kjs.2012.9.1.18, 25983783 PMC4432379

[4] Narain AS Hijji FY Markowitz JS Kudaravalli KT Yom KH Singh K. Minimally invasive techniques for lumbar decompressions and fusions. Curr Rev Musculoskelet Med. (2017) 10:559–66. doi: 10.1007/s12178-017-9446-z, 29027622 PMC5685968

[5] Pan M Li Q Li S Mao H Meng B Zhou F . Percutaneous endoscopic lumbar discectomy: indications and complications. Pain Physician. (2020) 23:49–56. 32013278

[6] Wang F Wang T Zhang S He L Chen K Zhou X . Is minimally invasive superior to traditional open technique for 2-segmental transforaminal interbody fusion in the treatment of lumbar degenerative disease? Clin Spine Surg. (2025). doi: 10.1097/BSD.0000000000001844, 40525712

[7] Song H Wang A Wang T Fan N Du P Wu Q . Percutaneous transforaminal endoscopic decompression versus posterior short-segment fusion for treating degenerative lumbar scoliosis with lumbar spinal stenosis: a cohort study with a minimum five year followup. Int Orthop. (2025) 49:1211–22. doi: 10.1007/s00264-025-06479-3 40063116, 40063116

[8] Zhang Y Zhu H Zhou Z Wu J Sun Y Shen X . Comparison between percutaneous transforaminal endoscopic discectomy and fenestration in the treatment of degenerative lumbar spinal stenosis. Med Sci Monit. (2020) 26:e926631. doi: 10.12659/MSM.926631, 33035202 PMC7552888

[9] Shi T Chen Z Hu D Li W Wang Z Liu W. Does type 2 diabetes affect the efficacy of therapeutic exercises for degenerative lumbar spinal stenosis? BMC Musculoskelet Disord. (2023) 24:198. doi: 10.1186/s12891-023-06305-0, 36927410 PMC10018869

[10] Udby PM Vestergaard T Ohrt-Nissen S Carreon LY. The impact of diabetes in patients with lumbar stenosis - a propensity-score matched study on patient-reported outcomes after surgery. Clin Neurol Neurosurg. (2023) 235:108038. doi: 10.1016/j.clineuro.2023.108038, 37949041

[11] Sun H Saeedi P Karuranga S Pinkepank M Ogurtsova K Duncan BB . IDF diabetes atlas: global, regional and country-level diabetes prevalence estimates for 2021 and projections for 2045. Diabetes Res Clin Pract. (2022) 183:109119. doi: 10.1016/j.diabres.2021.109119, 34879977 PMC11057359

[12] Park CH Min KB Min JY Kim DH Seo KM Kim DK. Strong association of type 2 diabetes with degenerative lumbar spine disorders. Sci Rep. (2021) 11:16472. doi: 10.1038/s41598-021-95626-y, 34389750 PMC8363740

[13] Lee CH Kim CH Chung CK Choi Y Kim MJ Yim D . Long-term effect of diabetes on reoperation after lumbar spinal surgery: a nationwide population-based sample cohort study. World Neurosurg. (2020) 139:e439–48. doi: 10.1016/j.wneu.2020.04.026, 32305613

[14] Arinzon Z Adunsky A Fidelman Z Gepstein R. Outcomes of decompression surgery for lumbar spinal stenosis in elderly diabetic patients. Eur Spine J. (2004) 13:32–7. doi: 10.1007/s00586-003-0643-7, 14614597 PMC3468040

[15] Kalff R Ewald C Waschke A Gobisch L Hopf C. Degenerative lumbar spinal stenosis in older people: current treatment options. Dtsch Arztebl Int. (2013) 110:613–23. doi: 10.3238/arztebl.2013.0613, 24078855 PMC3784039

[16] American Diabetes Association. Diagnosis and classification of diabetes mellitus. Diabetes Care. (2010) 33:S62–9. doi: 10.2337/dc10-S062 20042775 PMC2797383

[17] Ke P Han L Xu W Song Y Zhu B Wang L. Percutaneous endoscopic unilateral laminotomy and bilateral decompression improves gait quality and stance balance in patients with lumbar spinal stenosis: a retrospective cohort study. J Orthop Surg Res. (2025) 20:238. doi: 10.1186/s13018-025-05631-4, 40045396 PMC11881485

[18] Xuan A Ruan D Wang C He Q Wang D Hou L . Intradiscal injection of autologous discogenic cells in patients with discectomy: a prospective clinical study of its safety and feasibility. Stem Cells Transl Med. (2022) 11:490–503. doi: 10.1093/stcltm/szac013, 35427416 PMC9154349

[19] Zhu DC Lin JH Xu JJ Guo Q Wang YH Jiang C . An assessment of morphological and pathological changes in paravertebral muscle degeneration using imaging and histological analysis: a cross-sectional study. BMC Musculoskelet Disord. (2021) 22:854. doi: 10.1186/s12891-021-04734-3, 34625068 PMC8499494

[20] Zhu F Jia D Zhang Y Ning Y Leng X Feng C . Moderate to severe multifidus fatty atrophy is the risk factor for recurrence after microdiscectomy of lumbar disc herniation. Neurospine. (2023) 20:637–50. doi: 10.14245/ns.2346054.027, 37401083 PMC10323347

[21] Fan N Song H Zang L Wang A Wang T Yuan S . Clinical outcomes of percutaneous transforaminal endoscopic decompression for the treatment of degenerative lumbar scoliosis associated with spinal stenosis in elderly individuals: a matched comparison study. Int Orthop. (2024) 48:3197–205. doi: 10.1007/s00264-024-06318-x, 39320498

[22] Sakai Y Wakao N Matsui H Osada N Watanabe T Watanabe K. Insulin resistance as a risk factor for flavum hypertrophy in lumbar spinal stenosis. Spine Surg Relat Res. (2024) 8:583–90. doi: 10.22603/ssrr.2024-0025, 39659381 PMC11625720

[23] Lee CK Choi SK Shin DA Yi S Ha Y Kim KN . Influence of diabetes mellitus on patients with lumbar spinal stenosis: a nationwide population-based study. PLoS One. (2019) 14:e213858. doi: 10.1371/journal.pone.0213858, 30875413 PMC6420006

[24] Nagata K Nakamoto H Sumitani M Kato S Yoshida Y Kawamura N . Diabetes is associated with greater leg pain and worse patient-reported outcomes at 1 year after lumbar spine surgery. Sci Rep. (2021) 11:8142. doi: 10.1038/s41598-021-87615-y, 33854161 PMC8046758

[25] Illien-Junger S Grosjean F Laudier DM Vlassara H Striker GE Iatridis JC. Combined anti-inflammatory and anti-AGE drug treatments have a protective effect on intervertebral discs in mice with diabetes. PLoS One. (2013) 8:e64302. doi: 10.1371/journal.pone.0064302, 23691192 PMC3656842

[26] Fields AJ Berg-Johansen B Metz LN Miller S La B Liebenberg EC . Alterations in intervertebral disc composition, matrix homeostasis and biomechanical behavior in the UCD-T2DM rat model of type 2 diabetes. J Orthop Res. (2015) 33:738–46. doi: 10.1002/jor.22807 25641259, 25641259 PMC4408867

[27] Khan GM Chen SR Pan HL. Role of primary afferent nerves in allodynia caused by diabetic neuropathy in rats. Neuroscience. (2002) 114:291–9. doi: 10.1016/s0306-4522(02)00372-x, 12204199

[28] Ahlgren SC White DM Levine JD. Increased responsiveness of sensory neurons in the saphenous nerve of the streptozotocin-diabetic rat. J Neurophysiol. (1992) 68:2077–85. doi: 10.1152/jn.1992.68.6.2077, 1491258

[29] Fan Z Chen B Ding L Guo H. The causal association between type 2 diabetes and spinal stenosis: a Mendelian randomization analysis. Medicine (Baltimore). (2024) 103:e39894. doi: 10.1097/MD.0000000000039894, 39331863 PMC11441963

[30] Sun C Zhang H Wang X Liu X. Ligamentum flavum fibrosis and hypertrophy: molecular pathways, cellular mechanisms, and future directions. FASEB J. (2020) 34:9854–68. doi: 10.1096/fj.202000635R, 32608536

[31] Li S Du J Huang Y Gao S Zhao Z Chang Z . From hyperglycemia to intervertebral disc damage: exploring diabetic-induced disc degeneration. Front Immunol. (2024) 15:1355503. doi: 10.3389/fimmu.2024.1355503 38444852, 38444852 PMC10912372

[32] Yang F Zhu D Wang Z Ma Y Huang L Kang X . Role of advanced glycation end products in intervertebral disc degeneration: mechanism and therapeutic potential. Oxidative Med Cell Longev. (2022) 2022:7299005. doi: 10.1155/2022/7299005, 36573114 PMC9789911

[33] Wang F Wang Y Zhang S Pu M Zhou P. YTHDF2-dependent m(6)a modification of FOXO3 mRNA mediates TIMP1 expression and contributes to intervertebral disc degeneration following ROS stimulation. Cell Mol Life Sci. (2024) 81:477. doi: 10.1007/s00018-024-05503-w, 39625652 PMC11615171

[34] Wang L Valencak TG Shan T. Fat infiltration in skeletal muscle: influential triggers and regulatory mechanism. iScience. (2024) 27:109221. doi: 10.1016/j.isci.2024.109221, 38433917 PMC10907799

[35] D'Souza DM Al-Sajee D Hawke TJ. Diabetic myopathy: impact of diabetes mellitus on skeletal muscle progenitor cells. Front Physiol. (2013) 4:379. doi: 10.3389/fphys.2013.00379, 24391596 PMC3868943

[36] Miljkovic I Vella CA Allison M. Computed tomography-derived myosteatosis and metabolic disorders. Diabetes Metab J. (2021) 45:482–91. doi: 10.4093/dmj.2020.0277, 34352985 PMC8369205

[37] Zhang S Gu Z Ruan J Zhang J Chen H. Mitochondrial-targeted therapy for osteoarthritis: challenges and opportunities from basic research to clinical translation. Front Immunol. (2026) 17:1696120. doi: 10.3389/fimmu.2026.1696120, 41798918 PMC12963063

[38] Teichtahl AJ Urquhart DM Wang Y Wluka AE Wijethilake P O'Sullivan R . Fat infiltration of paraspinal muscles is associated with low back pain, disability, and structural abnormalities in community-based adults. Spine J. (2015) 15:1593–601. doi: 10.1016/j.spinee.2015.03.039, 25828477

[39] Tian X Zhao H Yang S Ding W. The effect of diabetes mellitus on lumbar disc degeneration: an MRI-based study. Eur Spine J. (2024) 33:1999–2006. doi: 10.1007/s00586-024-08150-8, 38361008

