# Supplementary material for: Clinical and imaging impact of diabetes mellitus on elderly patients with lumbar spinal stenosis: a retrospective propensity score-matched study with ≥5-year follow-up
Source: Front Med (Lausanne). 2026 May 29;13:1801937. doi: 10.3389/fmed.2026.1801937 (PMC13259833; doi:10.3389/fmed.2026.1801937)
Supplement: Supplementary file 1 [file Table_1.DOCX]

**Supplementary Table S1. Diabetes-related characteristics of patients in the matched DM group (n = 37).**

| **Variable** | **Value** |
| --- | --- |
| HbA1c (%) | 7.52 ± 0.39 |
| Duration of diabetes (years) | 13.89 ± 4.09 |
| Diabetes classification, n (%) |  |
| Type 1 diabetes mellitus | 0 (0.00) |
| Type 2 diabetes mellitus | 37 (100.00) |
| Antidiabetic treatment, n (%) |  |
| Oral hypoglycemic agents only | 21 (56.76) |
| Insulin only | 5 (13.51) |
| Diet control only | 1 (2.70) |
| Combined therapy | 10 (27.03) |
| Specific diabetes-related complications, n (%) |  |
| Diabetic peripheral neuropathy | 6 (16.22) |
| Diabetic retinopathy | 2 (5.41) |
| Diabetic nephropathy | 4 (10.81) |
| Diabetic macrovascular disease | 5 (13.51) |
