# Supplementary material for: Clinical and imaging impact of diabetes mellitus on elderly patients with lumbar spinal stenosis: a retrospective propensity score-matched study with ≥5-year follow-up
Source: Front Med (Lausanne). 2026 May 29;13:1801937. doi: 10.3389/fmed.2026.1801937 (PMC13259833; doi:10.3389/fmed.2026.1801937)
Supplement: Supplementary file 2 [file Table_2.DOCX]

**Supplementary Table S2. Correlation between HbA1c level and changes in clinical and imaging outcomes in the matched DM group.**

| **Variable** | **Correlation method** | **Correlation coefficient** | ***P* value** |
| --- | --- | --- | --- |
| ΔVAS-B score | Spearman | ρ = 0.021 | 0.900 |
| ΔVAS-L score | Spearman | ρ = -0.168 | 0.320 |
| ΔODI (%) | Pearson | r = -0.082 | 0.631 |
| ΔDHI (%) | Spearman | ρ = 0.446 | 0.006 |

Values are based on exploratory correlation analyses. Spearman rank correlation analysis was used for ΔVAS-B, ΔVAS-L, and ΔDHI, whereas Pearson correlation analysis was used for ΔODI. ΔVAS-B, ΔVAS-L, and ΔODI were calculated as the preoperative value minus the value at the last follow-up. ΔDHI was calculated as the preoperative DHI minus the DHI at the last follow-up. ρ indicates Spearman’s rank correlation coefficient; r indicates Pearson’s correlation coefficient.
